# Synthesis and Growth Mechanism of Stable Prenucleated (≈0.8 nm Diameter) PbS Quantum Dots by Medium Energy Ion Scattering Spectroscopy

**DOI:** 10.3390/ma12071109

**Published:** 2019-04-03

**Authors:** Young Ho Park, Seung Min Park, Kang-Won Jung, Yunju Hwang, Saurav Sorcar, Dae Woon Moon, Su-Il In

**Affiliations:** 1 Department of Energy Science and Engineering, DGIST, 333 Techno Jungang-daero, Hyeonpung-myeon, Dalseong-gun, Daegu 42988, Korea; nano.e.park@dgist.ac.kr (Y.H.P.); smpark01@dgist.ac.kr (S.M.P.); hunju1207@dgist.ac.kr (Y.H.); sorcar@dgist.ac.kr (S.S.); 2 Department of New Biology, DGIST, 333, Techno jungang-daero, Hyeonpung-myeon, Dalseong-gun, Daegu 42988, Korea; kw.jung@dgist.ac.kr

**Keywords:** PbS quantum dots, medium energy ion scattering (MEIS), non-classical nucleation theory, nucleation and growth, sub-nanometer

## Abstract

In the current work, stable prenucleated PbS quantum dots (QDs) with a sub-nanometer (0.8 nm) size have been successfully synthesized via a systematically designed experiment. A detailed analysis of critical nucleation, growth, and stability for such ultrasmall prenucleated clusters is done. The experimental strategy is based on controlled concentration, temperature and injection of respective precursors, thus enabling us to control nucleation rate and separation of stable sub-nanometer PbS QDs with size 0.8 nm. Significantly, by providing additional thermal energy to sub-nanometer PbS QDs, we achieved the fully nucleated cubic crystalline structure of PbS with size of around 1.5 nm. The size and composition of the prenucleated QDs are investigated by sophisticated tools like X-ray photoelectron spectroscopy (XPS) and medium energy ion scattering (MEIS) spectroscopy which confirms the synthesis of PbS with Pb^2+^ rich surface while the UV-Vis spectroscopy and X-ray diffraction (XRD) data suggests an alternative crystallization path. Non-classical nucleation theory is employed to substantiate the growth mechanism of prenucleated PbS QDs.

## 1. Introduction

Nanometer-scale research has captivated scientific interest, with an important aspect over the synthesis of nanoparticles as quantum dots and colloidal dispersions [1,2,3]. To date, a variety of novel synthetic methods, combination of advanced analytical tools, and theoretical approaches have been developed to explore and understand critical nucleation, vital for the controlled nucleation and growth of quantum nanomaterials including, but certainly not limited to, semiconductors such as CdS, CdSe, PbSe, ZnS, TiO_2_, and SnO_2_ [4,5,6,7,8,9]. Controlling the growth process of critical nuclei is within our grasp, but requires a new understanding regarding fundamental principles. It is crucial to understand the interdependence of the actual structure (morphology) and early-stage reactivity of the critical nuclei. Said differently, an important frontier of materials science is the ability to control sub-nanometer quantum dot crystal structures by the manipulation of synthetic conditions and critical nuclei atomic composition.

Quantum dots, ultrasmall nanoparticles, have received wide attention due to their unique and specific size-dependent properties [10,11]. In general, classical nucleation theory (CNT) is commonly used to explain the nucleation process of quantum dots with respect to kinetic and thermodynamic components [12]. Contrary to the well-recognized CNT, an alternative crystallization pathway (ACP) involving stable clusters prior to nucleation has been discovered [13,14], researchers have shown the prenucleation cluster pathway as a truly non-classical concept of nucleation.

Gebauer et al. [14] discovered formation of thermodynamically stable prenucleated clusters during CaCO_3_ nucleation, with an activation barrier that is negligible compared to thermal energy. In the solution phase, the state of prenucleated clusters represents a metastable minimum in free energy. Presumably this alternative mechanism of nucleation can be employed in the crystallization of various other materials with, we hypothesize, each material having its own unique nucleation pathway, like fingerprints or genes, and this intriguing prospect in turn motivating our research.

Use of MEIS spectroscopy as an analytical tool is vivid and is being utilized across various researches such as thin film analysis [15], interface quality improvement [16], and measurement of lattice deformation of strained heterogeneous epitaxial structures [17]. It has been used for exploring the structural properties of bimetallic nanoparticles, in order to analyze their composition and atomic arrangement [18]. Recently, Moon et al. [19] had successfully quantified calcium phosphate nanocluster growth using time of flight MEIS spectroscopy. Therefore, MEIS has become an important characterization tool for investigating and studying the growth mechanism of ultra-small nanoparticles. 

Herein we have investigated the critical nucleation and growth of lead sulphide (PbS), one of the extensively studied II–VI materials, promising for applications in the fields of photonic and optical devices [20], and so too solar cells [21,22,23]. PbS possesses several advantages that facilitate its use including ease of synthesis, relatively low cost, broad spectrum light absorption, tunable band gap energy, and strong quantum confinement with an exciton Bohr radius of approximately 20 nm [24]. In the current work, we study the growth processes of critical nuclei for PbS quantum dots (QDs) from prenucleation to their formation as well-defined nanoparticles, thus developing a new understanding of nucleation associated with non-classical theory.

The ability to synthesize prenucleated PbS QDs, described herein and which has not been previously reported, is an important step in the challenging task of understanding the underlying mechanism of critical nucleation and growth. We have developed a PbS QDs synthesis strategy using precursor pre-heating as well as precursor cold injection, as detailed in the sampling the product at various reaction times and temperatures. Employing the designed synthetic strategy, the nucleation rate is controlled to separate the prenucleated PbS QDs from each step of the nucleation process, as indicated by colour change, from bright yellow to red. Moreover, the size, stoichiometry and composition of the prenucleated PbS QDs are successfully determined by use of MEIS spectroscopy. To the best of our knowledge the synthesis and analysis of such sub-nanometer size PbS QDs have never been reported.

## 2. Materials and Methods

### 2.1. Materials

Lead oxide (PbO, 99.999%), 1-octadecene (ODE, technical grade, 90%), oleic acid (OA, technical grade, 90%), oleylamine (OAm, technical grade, 70%), and bis(trimethylsilyl) sulphide (TMS, [(Me_3_Si)]_2_S), were purchased from Sigma-Aldrich (Yougin, Korea) and used as received.

### 2.2. Synthesis of Prenucleated PbS Quantum Dots

All experimental procedures were performed under Ar atmosphere by using standard Schlenk line techniques. A PbO stock solution containing 0.45 g of PbO (2.016 mmol), 1.5 mL of oleic acid (4.75 mmol, OA), 0.165 mL of oleylamine (0.502 mmol, OAm) and 18 mL of 1-octadecene (ODE) was degassed in a 100 mL 3-neck flask at room temperature for 24 h under vacuum. The solution was then heated to 90 °C under Ar atmosphere and maintained at this temperature for 5 min until the solution colour changed to pure yellow indicating formation of lead oleate. At this stage the solution was cooled to ambient temperature to minimize the nucleation energy of the lead oleate. The sulphur precursor stock solution was prepared in 10 mL of ODE degassed in a 100 mL 3-neck flask at 80 °C for 24 h under vacuum. After degassing, 0.213 mL of bis(trimethylsilyl) sulphide (TMS, [(Me_3_Si)]_2_S) was added to the flask containing ODE and allowed to cool to ambient temperature. 0.5 mL of TMS stock solution was added drop wise to the previously prepared lead oleate solution by syringe. After 2 min of injection, the colour of the solution changed from yellow to orange, orange to reddish orange, reddish orange to bright red, and subsequently crimson red. The reaction was then continued at ambient temperature for 30 min, after which the reaction temperature was increased to 50 °C, 70 °C and 90 °C to analyse the particle growth at respective temperatures. Five different samples such as 30 °C (2 min), 30 °C (30 min), 50 °C (30 min), 70 °C (30 min) and 90 °C (30 min) were taken and purified with acetone and acetone/hexane via centrifugation at 15,000 rpm for 10 min each and finally dispersed in hexane.

### 2.3. Characterization

The absorption spectra of various samples are obtained by use of an ultraviolet visible near infrared (UV-Vis-NIR) spectrophotometer (Agilent Technologies, Santa Clara, CA, USA, Cary 5000). The samples are prepared in hexane. The X-ray diffraction (XRD) patterns for all samples obtained using X-Ray Diffractometer (Rigaku, Osaka, Japan, MiniFlex 600/300). The XRD samples are prepared by drop casting the respective sample (with known concentration in hexane) on the cleaned glass substrate followed by complete drying. Transmission electron microscope (TEM) images of PbS QDs are taken using a Hitachi HF-3300 FE-TEM (Hitachi, Tokyo, Japan), with an accelerating voltage of 300 kV. High resolution transmission electron microscope (HR-TEM) images of PbS QDs are taken using a Titan G2 ChemiSTEM Cs probe (FEI company, Hillsboro, OR, USA), with an accelerating voltage of 200 kV. PbS QDs samples are prepared by drop casting on a carbon coated Cu 200 mesh grid. X-ray photoelectron spectra (XPS) are obtained using an ESCALAB 250Xi (Thermo Fisher Scientific, Waltham, MA, USA), with each PbS QDs sample drop-cast onto a glass substrate in an Ar filled glove box. The binding energies obtained for the PbS QDs were calibrated based on the C 1s peak positioned at 284.5 eV as a reference [25,26]. MEIS spectra operated with He^+^ ion source accelerated at 80 keV are used to analyse PbS QDs samples casted on diamond-like carbon coated silicon substrates (DLC Si). MEIS data was analysed using POWERMEIS 2.0 simulation software.

## 3. Results

### 3.1. Morphology Analysis of Prenucleated PbS QDs

**Figure 1:**
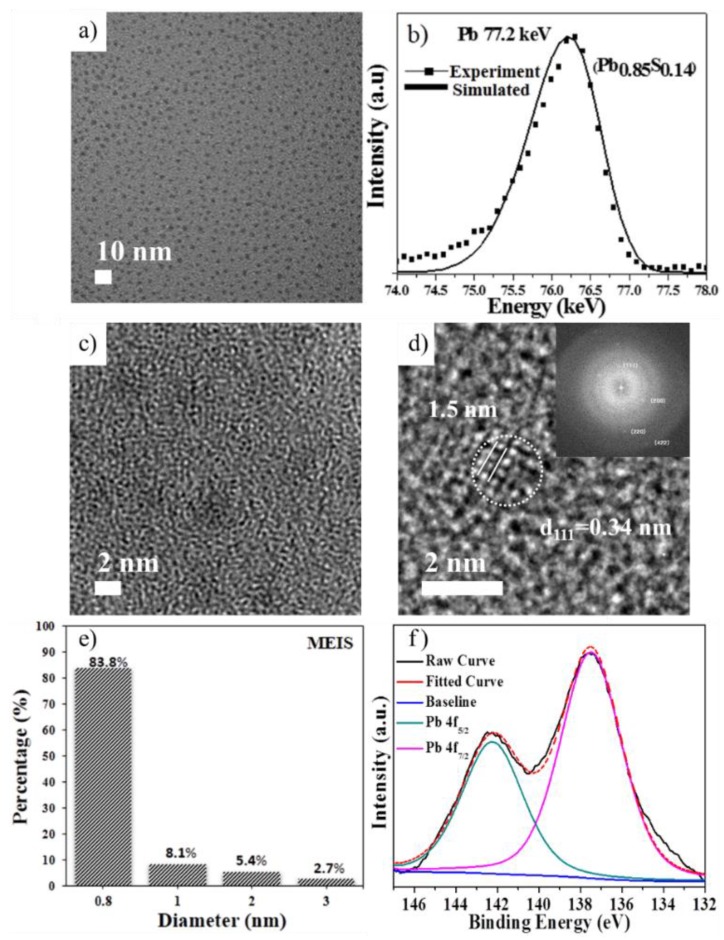
The prenucleated PbS QDs synthesized at 30 °C for 2 min: (**a**) TEM image, (**b**) MEIS spectra, and (**c**) HR-TEM image. (**d**) HR-TEM image of fully crystalized PbS QDs synthesized at 90 °C from the prenucleated PbS QDs. (**e**) shows the size distribution diagram obtained using MEIS of prenucleated PbS QDs with the molar ratio of Pb:S = 40:1 at 30 °C for 2 min, while (**f**) shows the Pb 4f XPS spectra.

The TEM image in Figure 1a shows the resulting uniform prenucleated PbS QDs (sample taken at 2 min after injection and at ambient temperature of 30 °C). It is difficult to obtain high resolution TEM images of the ultrasmall nanoparticles (see Figure 1c) due to inherent limitations of resolution and contrast without damaging the sample [27], as reported for various semiconductor or metal nanoparticles such as CdSe [28], PbSe [29], PbS [30] and Au [31]. Figure 1d shows a high resolution TEM image, and associated diffraction planes, of fully nucleated PbS QDs obtained at 90 °C. As indicated in Figure S1, the ultrasmall nanoparticles can be easily collapsed by highly focused electron beam energy [32]. For this reason, MEIS, which entails the use of dozens or hundreds of keV accelerated H^+^, He^+^ or Ne^+^ ions, has been employed in the present work. MEIS has been successfully used for characterization of the shape, size, composition, stoichiometry and size distribution of various nanoparticles structures such as Pt-Rh [33], CdSe-ZnS [34], Au [35], InAs-GaAs [36] and Au/Pd core-shell nanoparticles due to its excellent depth resolution [37], MEIS measurements average the strain profile over about 10^8^ dots [38], providing accurate information for quantum dots [38], nanoparticles [39,40] and nanoislands [41].

### 3.2. Physiochemical Property Analysis

MEIS analysis of prenucleated PbS QDs is shown in Figure 1b. The Pb signal appears at 77.2 keV of scattering energy in accordance with 80 keV He^+^ ion acceleration, which indicates the range of the Pb peak [33,34,35,36,37]. A tail in the lower energy is observed which is attributed to the aggregation of various sized particles on the diamond-like carbon coated silicon substrate (DLC Si). The particle size distribution calculated from the MEIS experimental data is about 0.8 nm (83.8%) as shown in Figure 1e. About 1.5% of the prenucleated PbS QDs are between 1.0~2.0 nm. The MEIS analysis exhibits a non-stoichiometric ratio of Pb and S, Pb:S = 6:1, a general phenomenon observed for presenting ultrasmall nanoparticles due to increased surface to volume ratio as compared to the bulk material [16], thus suggesting that the nucleation of PbS QDs does not follow the classical nucleation theory. Furthermore, XPS is used to investigate the chemical and electronic states. Figure 1f shows the Pb 4f core level spectra for prenucleated PbS QDs displaying the characteristic peaks of Pb 4f_7/2_ and Pb 4f_5/2_ associated with Pb–S bonds, appearing around a binding energy of 137.48 eV (FWHM: 3.57) and 142.9 eV (FWHM: 3.47), respectively [42]. The broadness of the peaks indicates linkage of Pb^2+^ with the oleic acid COO^−^ anion [42]. Our interpretation of the spectra is that it suggests the presence of a large amount of Pb^2+^ ions on the surface of the QDs, with their high surface to volume ratio. From the XPS analysis the ratio of Pb and S is found as 5.8:1, in excellent agreement with the value obtained from the MEIS analysis. The simulated and experimental peak result comparison shown in Figure S2 further confirms the consistency obtained between MEIS and XPS analysis. S 2s XPS was also conducted, and its presence in PbS QDs is shown in Figure S3. For each PbS quantum dots synthesized at different temperature, the peak area of Pb 4f and S 2s was calculated and the results are given in Table S1, supplementary information. Herein, it is difficult to analyze the exact stoichiometric ratio between Pb:S for each sample prepared at different temperature because of uncertainty of nucleation and growth.

**Figure 2:**
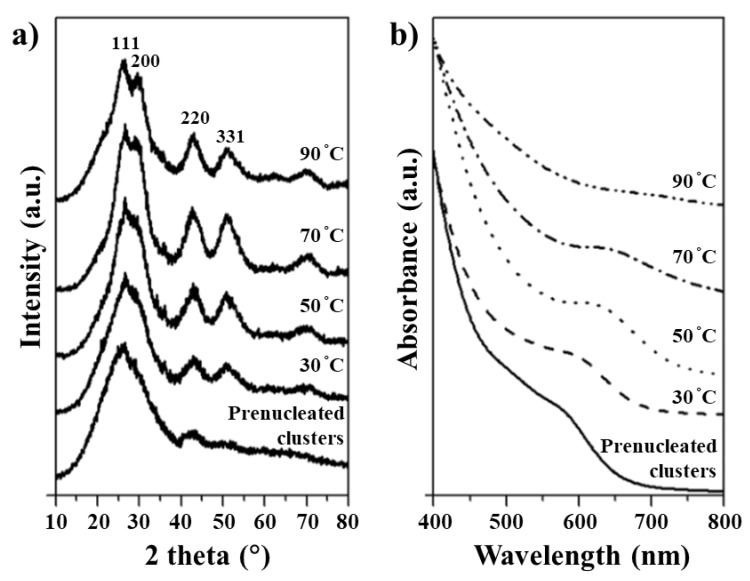
(**a**) XRD patterns, and (**b**) UV-Vis absorption spectra of prenucleated clusters as a function of precursor temperature, with the ratio of Pb:S = 40:1 at 30 °C for 2 min.

The growth of the prenucleated PbS QDs is studied by analysis of XRD patterns obtained for various aliquots taken during QDs growth with increasing temperature from 30 °C to 90 °C. One can see a clear transformation of amorphous phase to crystalline phase from the XRD patterns, Figure 2a, for various aliquots. The prenucleated PbS QDs show a broad peak in range of 2θ = 20–30° which can be associated to the combined (111) and (200) facets of PbS [43,44]. Presumably, at the beginning stages of nucleation, due to imperfect octahedral clusters and high surface to volume ratio, lead and sulphur ions have intense attraction resulting in the crystallization and thus formation of metastable clusters. Broad peak splitting is observed when the reaction temperature is increased above 70 °C, indicating the combination of imperfect octahedral prenucleated clusters (almost amorphous) and subsequent growth to a cubic crystalline structure due to thermal energy provided at higher reaction temperatures. Figure 1d shows a HR-TEM image and fast Fourier transform electron diffraction (FFT-ED) pattern (inset) for PbS QDs of 1.5 nm diameter (white circle) and cubic crystalline structure which corresponds well with XRD data. The UV-Vis absorption spectra of the QDs grown at different temperature are shown in Figure 2b. We make note of the UV-Vis peak broadening, which is consistent with earlier reports on PbS QDs [45,46,47,48]. It is observed that the first excitonic peak of the prenucleated PbS QDs [43,44] is blue shifted to 580 nm. However, when the reaction temperature is increased, after the appearance of prenucleated clusters, a redshift in absorption from 580 nm to 750 nm is observed which we assign to the growth of prenucleated clusters.

### 3.3. Growth Mechanism of Prenucleated PbS QDs

**Figure 3:**
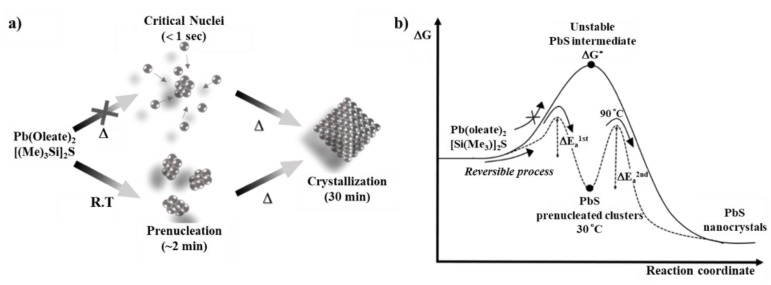
Schematic representation of (**a**) formation of nucleation and growth, (**b**) thermodynamics of non-classical nucleation theory for prenucleated PbS clusters.

The above results suggest an alternative crystallization pathway for the explanation of stable prenucleated PbS QDs that is different from the classical nucleation theory. Based on our experimental results we hypothesize a reversible process forming a stable prenucleated PbS clusters at ambient temperature (~30 °C) via variation of sulphur precursor concentration, nucleation rate and injection method as portrayed in Figure 3. Initially, when the S precursor is added drop wise at ambient temperature, the energy barrier i.e., the first ΔE_a_, is not very high and can be easily overcome by the lead sulphide precursors resulting in thermodynamically stable prenucleated PbS sub-nanometer particles. The formation of these prenucleated PbS QDs depends upon the stability of the precursors i.e., with less stable precursors the tendency to form metastable amorphous phase is more favored [49]. Simultaneously, as indicated by XRD data, PbS clusters can dissolve, resulting in an amorphous state of less energy that shows a semi-crystalline nature with reversible behaviour [49]. With increasing reaction temperature (90 °C), the prenucleated PbS rapidly nucleate following the trajectory of Ostwald ripening as displayed in Figure 3a,b. The nucleation rate at low temperatures, e.g., 30 °C, is slow and prenucleated clusters can be easily separated before crossing the second ΔE_a_. When the reaction temperature is increased the prenucleated PbS QDs and some semi-crystalline prenucleated clusters grow under Ostwald ripening, finally forming crystalline particles.

## 4. Conclusions

In conclusion, prenucleated clusters of lead sulphide are successfully synthesized by a specially designed temperature sensitive synthesis approach. Through the regulation of the precursor’s concentration, growth temperature, and injection method, it is possible for the dissolved ions to reach the stable prenucleated stage with appearance of sub-nanometer PbS QDs (≈0.8 nm). The PbS QDs possesses a non-stoichiometric ratio of Pb and S (Pb:S = 6:1) as determined by MEIS spectroscopy. We respectfully suggest that our synthesis technique and analysis strategy has the potential to significantly advance the science of nanomaterials.

## Supplementary Materials

The following are available online at https://www.mdpi.com/1996-1944/12/7/1109/s1, Figure S1: Correlation between detection limit and depth resolution for each analysis methods; Figure S2: Comparison between experimental and simulated MEIS spectra of prenucleated PbS QDs, and that of S (inset), using 80 keV accelerated He^+^ ions; Figure S3: XPS survey (a) and S 2s (b) spectra of prenucleated PbS QDs; Table S1: XPS data analysis for calculating Pb 4f and S 2s peak area of PbS QDs synthesized at different temperature.
