# Supplementary material for: Synthesis and Growth Mechanism of Stable Prenucleated (≈0.8 nm Diameter) PbS Quantum Dots by Medium Energy Ion Scattering Spectroscopy
Source: Materials (Basel). 2019 Apr 3;12(7):1109. doi: 10.3390/ma12071109 (PMC6479723; doi:10.3390/ma12071109)
Supplement: Supplementary file 1 [file materials-12-01109-s001.pdf]

# Supplementary Materials: Synthesis and Growth Mechanism of Stable Prenucleated (≈0.8 nm Diameter) PbS Quantum Dots by Medium Energy Ion Scattering Spectroscopy

**Young Ho Park [1,†], Seung Min Park [1,†], Kang-Won Jung [2], Yunju Hwang [1], Saurav Sorcar [1], Dae Woon Moon [2,*] and Su-Il In [1,*]**

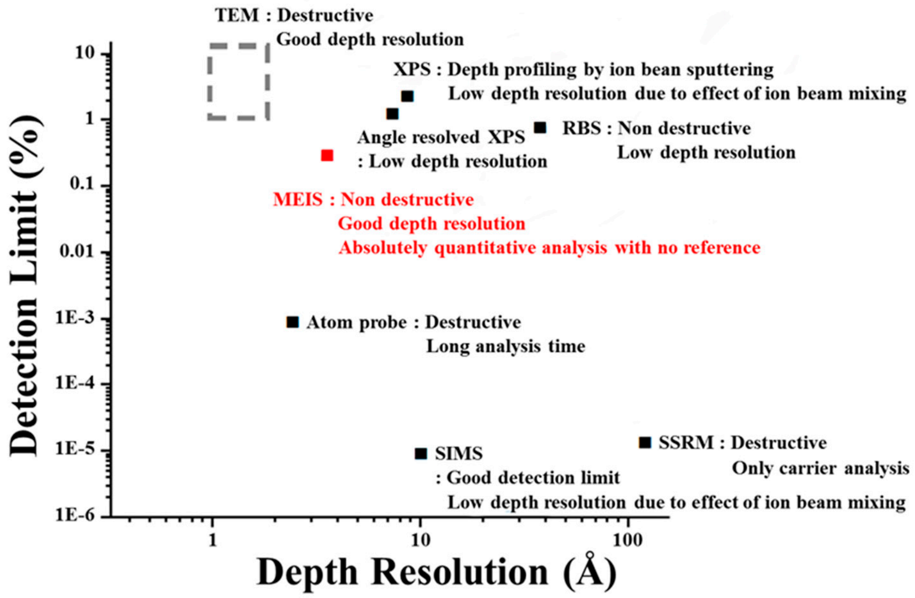


**Figure S1.** Correlation between detection limit and depth resolution for each analysis methods.

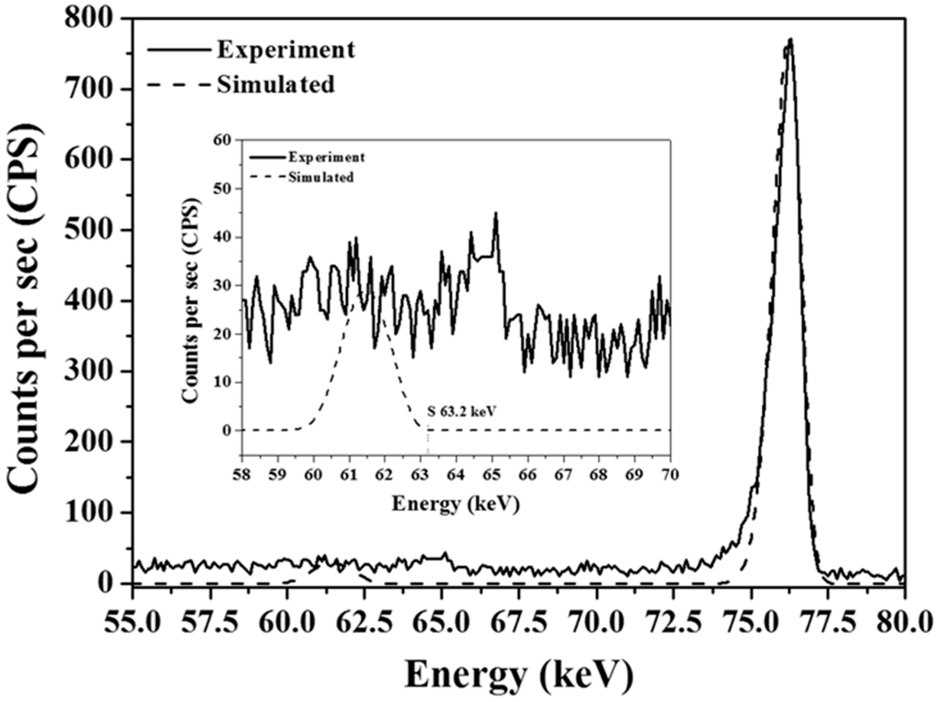


**Figure S2.** Comparison between experimental and simulated MEIS spectra of prenucleated PbS QDs, and that of S (inset), using 80 keV accelerated He⁺ ions.

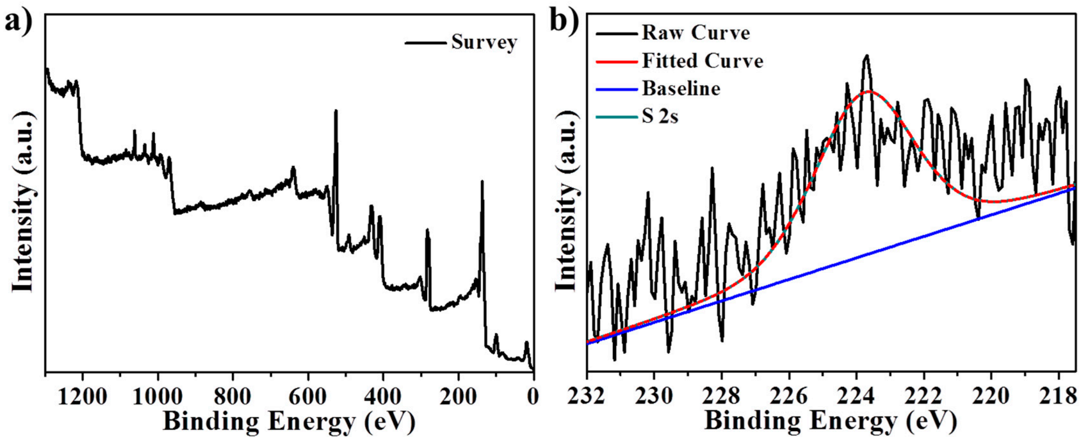


**Figure S3.** XPS survey (**a**) and S 2s (**b**) spectra of prenucleated PbS QDs.

**Table S1.** XPS data analysis for calculating Pb 4f and S 2s peak area of PbS QDs synthesized at different temperature.

| Condition | Element | Peak B.E. [a] (eV) | FWHM [b] | Peak Area |
|---|---|---|---|---|
| 30 °C, 30 min | Pb $4f_{7/2}$ | 137.51 | 2.3 | 4900 |
| | Pb $4f_{5/2}$ | 142.38 | 2.3 | 3700 |
| | S 2s | 224.5 | 3 | 119.54 |
| 50 °C, 30 min | Pb $4f_{7/2}$ | 137.4 | 2.4 | 5500 |
| | Pb $4f_{5/2}$ | 142.24 | 2.3 | 4300 |
| | S 2s | 224.5 | 3 | 141.31 |
| 70 °C, 30 min | Pb $4f_{7/2}$ | 137.5 | 2.4 | 5700 |
| | Pb $4f_{5/2}$ | 142.4 | 2.4 | 4400 |
| | S 2s | 224.5 | 3 | 148.6 |
| 90 °C, 30 min | Pb $4f_{7/2}$ | 137.38 | 2.3 | 5400 |
| | Pb $4f_{5/2}$ | 142.3 | 2.3 | 4400 |
| | S 2s | 224.5 | 3 | 149.27 |

[a]: B.E. = Binding Energy; [b]: FWHM = Full Width Half Maximum
